# International survey on skin patch test procedures, attitudes and interpretation

**DOI:** 10.1186/s40413-016-0098-z

**Published:** 2016-03-04

**Authors:** Luciana K. Tanno, Razvigor Darlenski, Silvia Sánchez-Garcia, Matteo Bonini, Andrea Vereda, Pavel Kolkhir, Dario Antolin-Amerigo, Vesselin Dimov, Claudia Gallego-Corella, Juan Carlos Aldave Becerra, Alexander Diaz, Virginia Bellido Linares, Leonor Villa, Lanny J. Rosenwasser, Mario Sanchez-Borges, Ignacio Ansotegui, Ruby Pawankar, Thomas Bieber, on behalf of the WAO Junior Members Group

**Affiliations:** Hospital Sírio Libanês and Post-graduation Program in Health Sciences of IAMSPE, Rua Prof Arthur Ramos, 183, cj 21 01454-011, São Paulo, SP Brazil; Department of Dermatolgy and Venereology, Tokuda Hospital Sofia, Sofia, Bulgaria; Allergy Department. Hospital Infantil Universitario Niño Jesús, Madrid, Spain; Department of Public Health and Infectious Diseases, “Sapienza” University of Rome, Rome, Italy; Allergy Clinic, Paris, 75015 France; Department of Dermatology and Venereology, Sechenov First Moscow State Medical University, Moscow, Russia; Immune System Diseases and Oncology Service-Allergy Unit. Hospital Universitario Príncipe de Asturias, Medicine and Medical Specialities Department, Universidad de Alcalá. Alcalá de Henares, Madrid, Spain; Department of Allergy and Immunology, Cleveland Clinic Florida, 2950 Cleveland Clinic Blvd Weston, Florida, FL 33331 USA; Medicine Faculty, Xochicalco University, Tijuana, Mexico; Allergy and Immunology Division, Hospital Nacional Edgardo Rebagliati Martins, Lima, Peru; Department of Allergy, Medical Center, Guira de Melena, Havana, Cuba; Intercenter Allergy Unit, Virgen Macarena University Hospital, Seville, Spain; Sanatorio Privado San Roque, Marcos Juárez, Córdoba Argentina; Department of Pediatrics, Division of Immunology Research, Children’s Mercy Hospitals & Clinics, Kansas City, MO 64108 USA; Allergy and Clinical Immunology Department, Centro Medico Docente La Trinidad, Caracas, Venezuela; Department of Allergy and Immunology, Hospital Quirón Bizkaia, Bizkaia, Spain; Division of Allergy, Department of Pediatrics, Nippon Medical School, Tokyo, Japan; Department of Dermatology and Allergy, Rheinische Friedrich-Wilhelms-University Bonn, Bonn, Germany

**Keywords:** Allergy, Contact dermatitis, Sensitization, Skin patch test, Survey

## Abstract

**Background:**

Skin patch test is the gold standard method in diagnosing contact allergy. Although used for more than 100 years, the patch test procedure is performed with variability around the world. A number of factors can influence the test results, namely the quality of reagents used, the timing of the application, the patch test series (allergens/haptens) that have been used for testing, the appropriate interpretation of the skin reactions or the evaluation of the patient’s benefit.

**Methods:**

We performed an Internet –based survey with 38 questions covering the educational background of respondents, patch test methods and interpretation. The questionnaire was distributed among all representatives of national member societies of the World Allergy Organization (WAO), and the WAO Junior Members Group.

**Results:**

One hundred sixty-nine completed surveys were received from 47 countries. The majority of participants had more than 5 years of clinical practice (61 %) and routinely carried out patch tests (70 %). Both allergists and dermatologists were responsible for carrying out the patch tests. We could observe the use of many different guidelines regardless the geographical distribution. The use of home-made preparations was indicated by 47 % of participants and 73 % of the respondents performed 2 or 3 readings. Most of the responders indicated having patients with adverse reactions, including erythroderma (12 %); however, only 30 % of members completed a consent form before conducting the patch test.

**Discussion:**

The heterogeneity of patch test practices may be influenced by the level of awareness of clinical guidelines, different training backgrounds, accessibility to various types of devices, the patch test series (allergens/haptens) used for testing, type of clinical practice (public or private practice, clinical or research-based institution), infrastructure availability, financial/commercial implications and regulations among others.

**Conclusion:**

There is a lack of a worldwide homogeneity of patch test procedures, and this raises concerns about the need for standardization and harmonization of this important diagnostic procedure.

## Background

Skin patch test (PT) is an essential in vivo test procedure to confirm T-lymphocyte-mediated allergic diseases and/or sensitization in subjects with allergic contact dermatitis, atopic eczema, as well as food and drug allergies. It provides evidence of sensitization and can confirm the etiological diagnosis of a suspected type IV allergy by reproducing a local allergic reaction on a small area, where the diluted test substances are placed. In cases in which contact urticarial syndrome is suspected, it is also been used to explore direct type (urticarial) reaction by performing 20 min reading. It is a non-invasive, rather simple method, but the allergen selection, the proper allergen concentration and the interpretation of the results require expertise. It can be reproducible when carried out by trained health professionals [1–4].

Although during the last decades great efforts have been devoted to optimization and standardization of the patch testing materials and methodology [5–19], the value of this test depends on whether the clinical presentation warrants its use, the quality of reagents used, the timing of the application, an appropriate interpretation of the reaction and the relevance for the patient’s benefit.

The procedure of PT still largely resembles the original methods described; however, a wide array of interpretations and modifications has led to diminished comparability when PT results are reported by different observers. This may be influenced by different professional background training (allergy/immunology, dermatology, pediatrician or other), type of clinical practice (private, public, clinical or research-based institution), accessibility to various guidelines and different types of devices, recommendations of the different National Society, among others.

To better appraise the many different PT methods and forms of interpretation in use worldwide and to contribute to the harmonization of its technique for a more rational comparison of their results, the WAO Junior Members Group (WAO JMG) conducted a first survey among members of WAO, and representatives responding on behalf of the national Member Societies of WAO, and the WAO JMG.

## Methods

A web-based questionnaire was constructed and circulated among the members of the WAO JMG Steering Committee (July-August 2013). The final version comprised a total of 38 questions covering the professional background of respondents, PT methods and interpretation of the results [1–35]. The survey used a skip logic pattern, allowing participating physicians to avoid certain sections according to their responses in preceding questions. The questions were presented in a fixed order and most of them were close-ended. Most of the questions were designed to be answered in a compulsory manner. The survey was then beta tested by the WAO JMG and WAO headquarters before being sent out.

The protocol was approved by the WAO Executive Committee and Board of Directors (November 2013) and launched by e-mail by the WAO headquarters to all representatives of, WAO Member Societies, and members of the WAO JMG, regardless of the specialty, affiliation, or nationality (December 2013). We sent out an introduction letter containing a link (Internet address) to the online questionnaire that was unique to each participating member. Two reminders were sent (January 2014 and March 2014) and all the respondents were given 90 days to reply.

The data were recorded in SPSS for Windows v.22. Analyses of the difference in frequencies across groups were performed with the Pearson Chi-squared test and a *p* value ≤0.05 was considered significant. Cramer’s V was used for the evaluation of the strength of statistically significant associations.

## Results

**Fig. 1:**
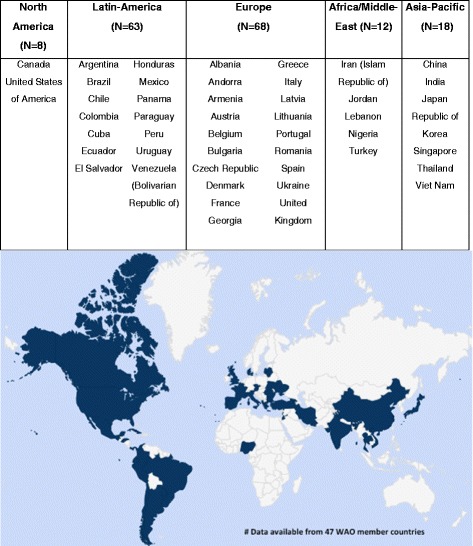
Number of responses, list and distribution of countries that participated in the survey

We received a total of 169 completed surveys from 47 countries of within regions of WAO member Societies: Africa/Middle-East (AME), Asia-Pacific (AP), Europe (EU), Latin-America (LA) and North America (NA) (Fig. 1).

**Fig. 2:**
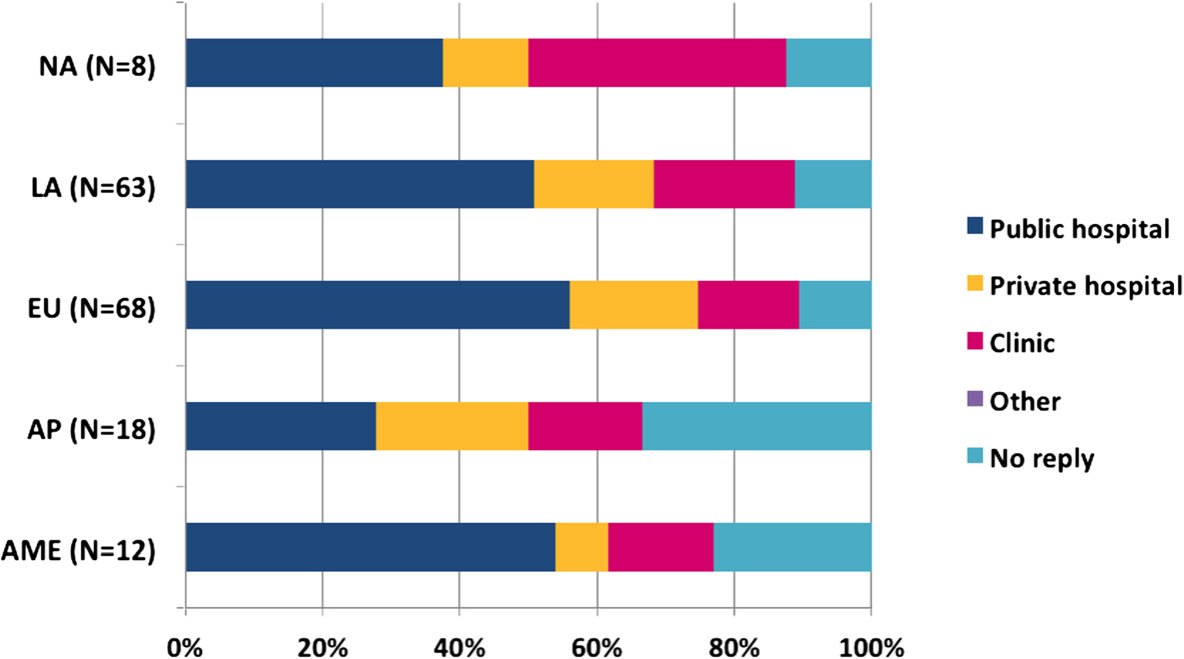
Clinical practices of the survey responders. Africa/Middle-East (AME), Asia-Pacific (AP), Europe (EU), Latin-America (LA) and North America (NA)

Among all responders, 52 % replied as members of the WAO JMG and 48 % on behalf of their national society, a Member Society of WAO. (Fig. 1). Most of the participants indicated allergy (76 %) as their main specialty (according to the respective national educational requirements), followed by clinical immunology (8 %) and dermatology and pediatrics (both 4 %). The majority of participants had more than 5 years of clinical practice (61 %) and routinely carried out PT (70 %), more than once per week (47 %) mainly in public hospitals (Table 1 and Fig. 2).

**Table 1:** Characteristics of survey responders

Characters of responders	Regions of WAO member societies				
	AME (*N* = 12) (%)	AP (*N* = 18) (%)	EU (*N* = 68) (%)	LA (*N* = 63) (%)	NA (*N* = 8) (%)
**Groups**					
WAO JM (*N* = 88)	5 (42)	8 (45)	40 (59)	32 (51)	3 (38)
WAO member society representative (*N* = 81)	7 (58)	10 (55)	28 (41)	31 (68)	5 (62)
**Specialty**					
Allergist (*N* = 128)	7 (58)	10 (56)	56 (82)	47 (75)	8 (100)
Clinical Immunologist (*N* = 14)	3 (25)	0 (0)	2 (3)	9 (13)	0 (0)
Pediatrician (*N* = 7)	0 (0)	2 (11)	4 (6)	1 (2)	0 (0)
Dermatologist (*N* = 6)	0 (0)	2 (11)	3 (5)	1 (2)	0 (0)
Other clinical specialty (*N* = 5)	2 (17)	2 (11)	2 (3)	5 (8)	0 (0)
Researcher (*N* = 3)	0 (0)	2 (11)	1 (1)	0 (0)	0 (0)
**Years of practice**					
<1 year (*N* = 12)	0 (0)	0 (0)	4 (6)	5 (8)	3 (37)
1 to 5 years (*N* = 54)	5 (42)	5 (28)	21 (31)	22 (35)	1 (13)
5 to 10 years (*N* = 40)	2 (16)	3 (17)	28 (41)	5 (8)	2 (25)
>10 years (*N* = 63)	5 (42)	10 (55)	15 (22)	31 (49)	2 (25)
**Routinely carry out PT**					
Yes (*N* = 119)	6 (50)	10 (56)	49 (72)	48 (76)	6 (75)
No (*N* = 50)	6 (50)	8 (37)	19 (28)	15 (24)	2 (25)
**Use any guideline**					
Yes (*N* = 99/119)	6 (100)	10 (100)	40 (82)	38 (79)	5 (84)
No (*N* = 20/119)	0 (0)	0 (0)	9 (18)	10 (21)	16 (16)
**Number of PT/week**					
<1/week (*N* = 56)	2 (17)	6 (33)	20 (29)	23 (36)	5 (63)
1–10/week (*N* = 79)	5 (42)	6 (33)	37 (55)	29 (46)	2 (25)
11–25/week (*N* = 5)	1 (4)	0 (0)	2 (3)	2 (3)	0 (0)
26–50/week (*N* = 3)	1 (4)	0 (0)	1 (1)	1 (2)	0 (0)
>50/week (*N* = 4)	0 (0)	1 (6)	0 (0)	3 (5)	0 (0)
No reply (*N* = 22)	3 (25)	5 (28)	8 (12)	5 (8)	1 (12)

Africa/Middle-East (AME), Asia-Pacific (AP), Europe (EU), Latin-America (LA) and North America (NA)

The PT was mostly used for clinical aims (61 %), but 23 % of the responders used the procedure for both clinical and research goals, while 3 % confine the use for research only. Both allergists and dermatologists were responsible for carrying out PT in different countries as informed by 43 % responses, but in Asian-Pacific countries the number of dermatologists performing PT predominated (Table 2).

**Table 2:** Properties of the skin patch test practice

Attitudes of the skin patch test practice	Regions of WAO member societies				
	AME (*N* = 12) (%)	AP (*N* = 18) (%)	EU (*N* = 68) (%)	LA (*N* = 63) (%)	NA (*N* = 8) (%)
**Who performs PT?**					
Allergists (*N* = 63)	3 (25)	5 (28)	22 (32)	32 (51)	1 (12)
Dermatologists (*N* = 29)	2 (17)	9 (50)	7 (10)	10 (16)	1 (12)
Both (*N* = 72)	7 (58)	3 (17)	37 (54)	19 (30)	6 (76)
Nobody (*N* = 3)	0 (0)	1 (5)	0 (0)	2 (3)	0 (0)
Other (*N* = 3)	1 (8)	0 (0)	2 (3)	0 (0)	0 (0)
**To whom PT is performed?**					
Adults (N = 45)	3 (25)	5 (28)	29 (43)	7 (11)	1 (12)
Children (N = 19)	4 (33)	0 (0)	5 (7)	9 (14)	1 (12)
Both (N = 83)	3 (25)	7 (39)	26 (38)	41 (65)	6 (76)
No reply (N = 22)	2 (17)	6 (33)	8 (12)	6 (10)	0 (0)
**Formulations tested**					
Commercial formulations (N = 62)	3 (25)	7 (39)	26 (38)	24 (38)	4 (50)
Home-made preparations (N = 32)	2 (17)	2 (11)	6 (9)	20 (32)	0 (0)
Both (N = 48)	5 (42)	1 (5)	26 (38)	12 (19)	0 (0)
No reply (N = 31)	2 (17)	8 (44)	10 (15)	7 (11)	4 (50)
**Data in healthy controls for home-made preparation?**					
Yes (N = 55)	3	3	28	21	0
No (N = 50)	5	7	14	20	4
**Kind of chambers used**					
Plastic (N = 50)	5 (42)	5 (28)	23 (34)	19 (30)	1 (12)
Aluminum (N = 49)	1 (8)	2 (11)	15 (22)	30 (48)	1 (12)
Pre-loaded with allergens (N = 26)	1 (8)	2 (11)	10 (15)	5 (8)	3 (38)
On water-proof plasters (N = 14)	1 (8)	3 (17)	11 (16)	0 (0)	0 (0)
Other (N = 3)	0 (0)	0 (0)	0 (0)	2 (3)	1 (12)
No reply	4 (33)	6 (33)	9 (13)	7 (11)	2 (26)

Africa/Middle-East (AME), Asia-Pacific (AP), Europe (EU), Latin-America (LA) and North America (NA)

Although 37 % indicated the use of commercial formulations, 28 % used both home-made preparations and commercialized formulations and 19 % home-made preparations exclusively (Table 2). Thirty percent of physicians indicated having no data on home-made preparations use in healthy controls or exposed to different substances. The participants who repeated the PT used more often commercial formulations (48.5 %) than home-made (13.6 %) or both (37.9 %) (*χ*2 = 5.980, *p* = 0.05, V = 0.209).

The most used chambers were made of plastic (30 %) and aluminum (29 %), and geographical differences could be appreciated, as chambers with pre-loaded allergens and water-proof plaster chambers were in use mainly in Europe (Table 2).

**Fig. 3:**
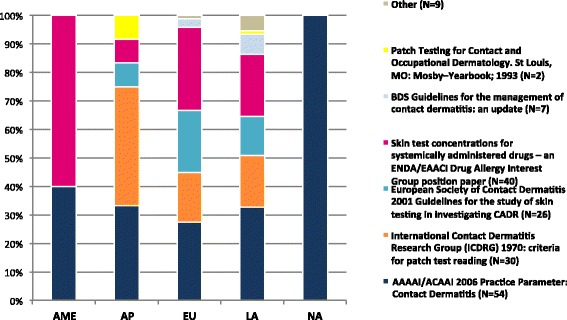
Guidelines in use across the regions of WAO member Societies (more than one option per responder was permitted) Africa/Middle-East (AME), Asia-Pacific (AP), Europe (EU), Latin-America (LA) and North America (NA)

Of all 119 participants who routinely performed PT, 83 % used clinical guidelines. The most widely used clinical practice guideline was the AAAAI/ACAAI 2006 Practice Parameter: Contact Dermatitis (32 %), followed by the Skin test concentrations for systemically administered drugs – an ENDA/EAACI Drug Allergy Interest Group position paper (24 %), the International Contact Dermatitis Research Group (ICDRG) 1970: criteria for patch test reading (18 %). North American physicians use exclusively the AAAAI/ACAAI 2006 Practice Parameter: Contact Dermatitis. The remaining guidelines used are summarized in Fig. 3. Professionals who routinely used guidelines in their clinical practice were those who more often conducted PT (87.4 vs 12.6 %, *χ*2 = 17.373, *p* < 0.0005, V = 0.361) and more frequently performed retesting (90.5 vs 9.5 %, *χ*2 = 3.804, *p* = 0.05, *V* = 0.174).

Professionals who routinely carried out PT (more than one PT per week) were those who used guidelines in the clinical practice (90.7 vs 9.3 %, *χ*2 = 8.420, *p* = 0.004, V = 0.254) and for clinical purpose (65.1 vs 34.9 %, *χ*2 = 6.465, *p* = 0.011, V = 0.216).

**Fig. 4:**
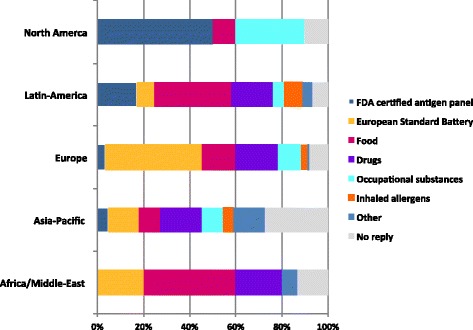
Main groups of substances used in skin patch tests worldwide (more than one response/participant permitted) Africa/Middle-East (AME), Asia-Pacific (AP), Europe (EU), Latin-America (LA) and North America (NA)

The main groups of the substances tested were those in the European Standard Battery (42 %), food (36 %) and drugs (28 %). Occupational substances were tested more frequently in North America, Europe and Asia-Pacific and FDA certified allergen panel was in use mainly in North and Latin America (Fig. 4).

**Fig. 5:**
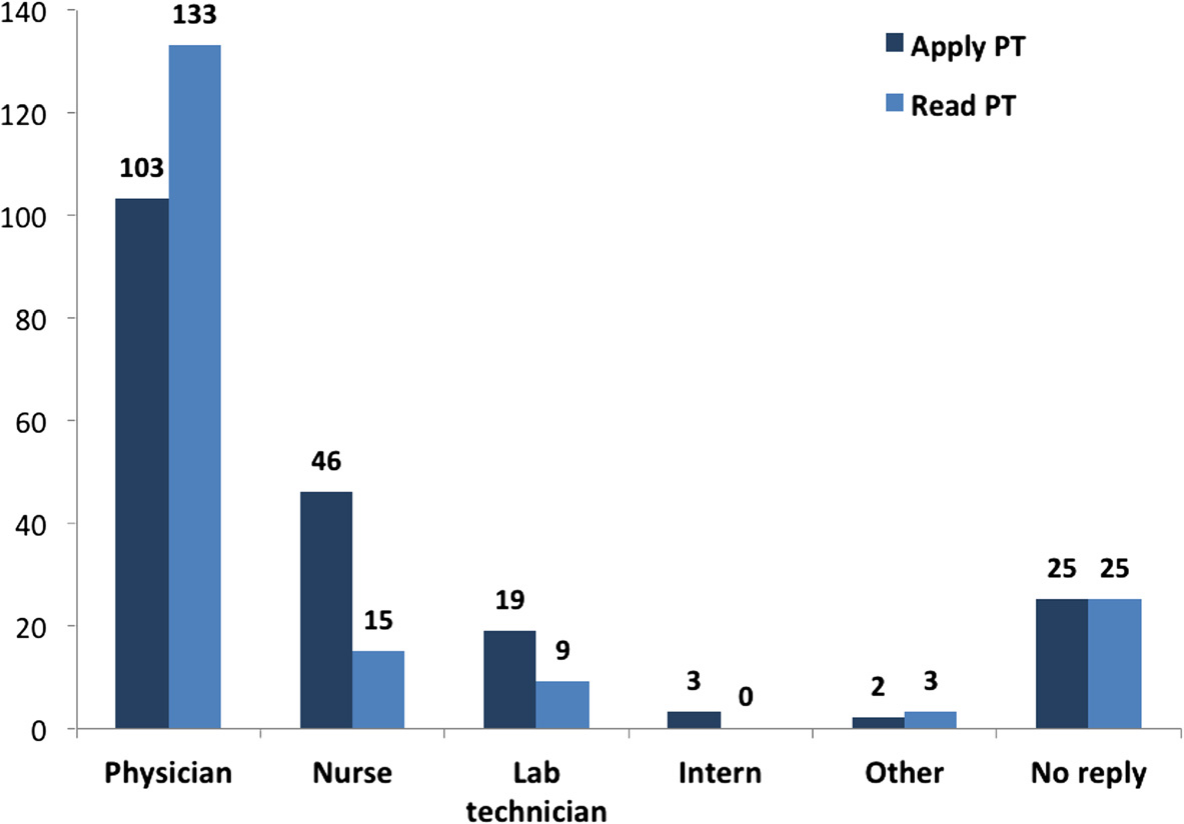
Who is responsible for applying and reading skin patch test in your current practice?

As indicated in Fig. 5, physicians were responsible for applying (61 %) and reading the PT results (78 %). However, as indicated by 16 % of repliers, the PT reading was performed by a non-physician.

**Fig. 6:**
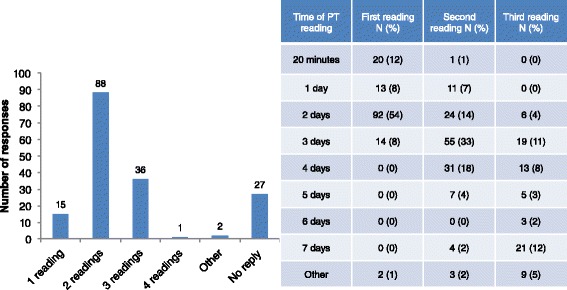
Number and time of the skin patch test readings

Seventy-three percent of the responders usually did 2 or 3 readings of PT. The first reading was mainly performed after 2 days (54 %), but 12 % did the first reading after 20 min of the PT application. Thirty-three percent did the second reading after 3 days and 18 % after 4 days of the test implementation. The third reading was performed by 76 responders, mainly after a week (Fig. 6). The majority (68 %) of responders pointed out the need of the same person reading the results of the test at different times.

**Fig. 7:**
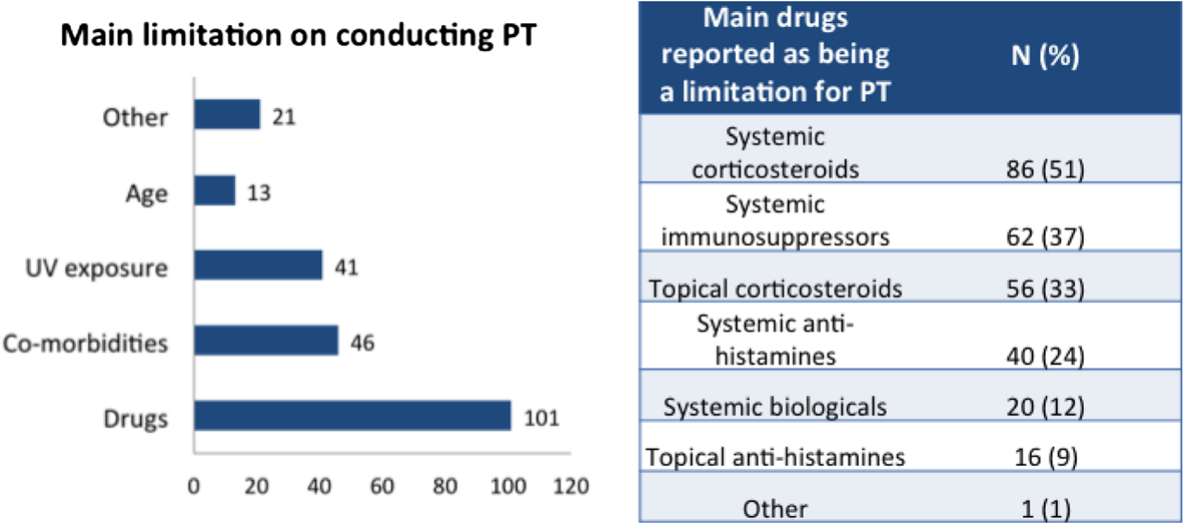
Main limitations to perform the skin patch test (PT)

**Fig. 8:**
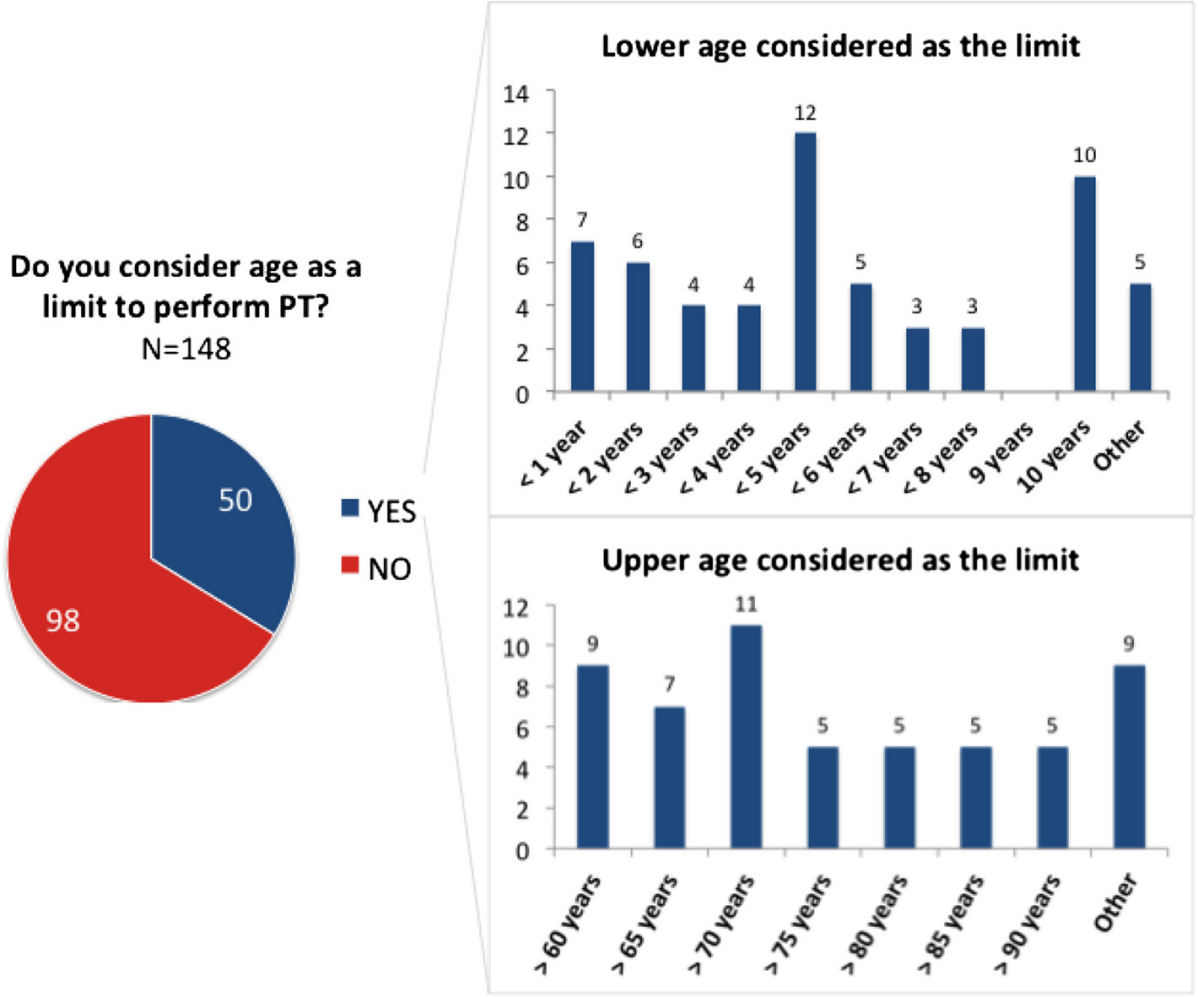
Age limitation to perform the skin patch test (PT)

Figure 7 lists the main limitations on conducting PT. Systemic and topical corticosteroids and immunosuppressants were indicated as being the main drugs able to interfere in PT results, however, systemic and topical anti-histamines were still reported as being limitations for the procedure (Fig. 7). Of all 148 responders, 50 (34 %) considered age as a limitation to perform the PT. Most of those participants pointed out “less than 5 years” as being the lower limit of age and “more than 70 years” as being the upper limit of age (Fig. 8).

Pruritus (72 %), exacerbation of the primary local reactions (29 %) and erythroderma (12 %) were listed as the main adverse reactions. The professionals who perform more than 1 PT per week observed more often adverse reactions than those who did less than 1 patch test per week (86.7 vs 13.3 %, *χ*2 = 6.416, *p* = 0.04, V = 0.214).

Out of 142 replies, 30 % of members asked for a consent form before conducting the PT and 78 % provided an allergen information document or an allergy passport in case of positive result. The results of PT were recorded into the own database by 53 % of respondents, while 29 % stored the results into the Hospital database. None of the respondents reported the use of an International collaboration database.

## Discussion

The WAO JMG initiative on collecting data regarding Skin Patch Test Procedures and Interpretation is the first international survey ever conducted in the Allergy field to review details of this procedure. This study demonstrates the current ubiquity of the PT use in daily clinic worldwide and underlines a clear need for harmonization of PT procedures.

International surveys represent one of the most feasible methods for obtaining relevant information from professionals and the online basis can contribute to collect a higher number of responders from all around the world in a short period of time. Considering the period of time spent to capture the replies, this survey was successful on reaching a high number of professionals, both WAO JMG and responders on behalf of the National Member Societies of WAO (Table 1), which may reflect a significant interest in this diagnostic procedure among all generations of different specialties. The responders performing PT in clinical practice were mainly clinicians with more than 5 years of professional experience.

The fact that there were more respondents from EU and AME regions may reflect the higher number of WAO members in these regions. On the other hand, we had no feedback from countries in which PT devices were not commercially available at the time of the survey, such as in the Russian Federation.

The heterogeneity of PT practice (Table 2 and Fig. 2) and the diversity of substances tested (Fig. 4) may be influenced by the level of guidelines awareness, different training backgrounds (allergy/immunology, dermatology, pediatric), accessibility to various types of devices, type of clinical practice (public or private practice, clinical or research-based institution), practice infrastructure availability, financial/commercial implications, and National regulations among others. The type of clinical practice (public or private practice, clinical or research-based institution) may influence in the infrastructure availability, the number of patients tested per day, the availability of patch test series (allergens/haptens) and in the referral patterns and, therefore, impacts in the SPT interpretation.

The WAO position paper published in 2008, stated the definition of Allergy as specialty when a physician who has successfully completed both a specialized training period in allergy and immunology and a training period in either internal medicine, or a sub-specialty of internal medicine such as dermatology, pneumology, or otorhinolaryngology, and/or pediatrics [36]. National educational requirements and curricula to become an allergist may vary from this definition. Although the allergy specialty is considered as a sub-specialty in some countries, in the current document we decided for considering as “allergist”, all who completed the degree following the above statement regardless of the previous formation.

Although the majority (68 %) of responders pointed out the need of the same person being responsible for subsequent readings at the different time points, Fig. 5 indicates that the PT is read by different types of health professionals. Therefore, we believe that all the professionals reading the tests may require specific training to avoid difficulties in interpretation and therefore diagnostic mistakes.

The majority of participants performed two readings of PT, however, this topic seems to be still controversial, since 36 % indicated to conduct one or three readings (Fig. 6). This discrepancy may be influenced by the type of substance tested, the suspected underlying mechanism (T-lymphocyte-mediated or direct type urticarial reaction), the different professional experience and the use of different guidelines, even though, these data underline the deficiency of interpretation standard, which can directly impact in the diagnosis. Besides, the lack of studies addressing the issue of quality management/issues in the field of PT with regards of training of the reader supports the need of a good quality education and formation actions.

Most of the responders indicated having patients with adverse reactions, including erythroderma; however, only 30 % of members currently complete an informed consent form before conducting the PT, with potential legal implications for the physicians. Believing that this situation has to improve, the WAO JMG may work on a consent form to be endorsed by the Allergy Academies to protect both professionals and patients.

The use of home-made preparations was indicated by 47 % of participants, what is expected since many substances are not available in standardized test panels. The absence of data in healthy controls for home-made preparations (Table 2) emphasizes the lack of standardization of these tests, which can increase the false-positive results as well as the adverse reactions. It highlights the need of procedure standardization and asking for a consent form.

Although our understanding of the immune system’s functions has changed substantially over the last 20 years, the presented data still suggest a lack of full understanding of the underlying immune mechanisms of contact dermatitis exemplified by the Fig. 7, in which systemic and topical anti-histamines were still reported as being limitations for the PT. In general, histamine has a limited importance in the pathophysiology of this disorder and these drugs are mainly used to smooth the symptom of pruritus. For this reason, scientific data don’t consider these drugs as limitations or contra-indications for the procedure. This data also bring up the need of implementing education tools to strengthen a better information for the new generation of health professionals working in the field.

The most important purpose of a guideline is to establish a statement used to determine a course of action, which is neither biding nor enforced, but aims to streamlines particular processes according to a routine. Many guidelines have emerged to outline medical diagnostic procedures, such as the PT [7–19] and many different Societies are making efforts to generate comparison data by multicentric studies. However, taking into account the current actions of Allergy Academies to promote the recognition of Allergy and Clinical Immunology as a specialty and to strengthen the awareness of allergic diseases around the world [37–39], we believe that it is now the best moment to review some procedures in use in the daily practice to identify gaps in which we can work on. The results of this survey highlight the heterogeneity of attitudes and practices on using the PT and clearly consolidate the current lack of standardization of PT methods universally accepted. We could observe the use of many different guidelines regardless to the geographical distribution (Fig. 3), the use of home-made preparations and different time of readings.

The present study has some limitations. We are aware of the possibility of a selection bias within large countries/regions, the president/chair of the WAO Member Society may not have had ample opportunity to run the survey through all members from different states/districts within the consultation period, especially where practices maybe heterogeneous and in large countries/regions. It was also not possible to access the response rate, as we are not aware of how many recipients got the survey email and the proportion of e-mails bounced by the online server. The definition concepts of some terms, such as for “erythroderma”, used worldwide were not accessed since it was not the aim of the current manuscript. In the current survey, we didn’t explore specific data such as application time or time of readings, per specific agents.

## Conclusion

We strongly believe that the results of this WAO JMG project underline a clear need of updating the PT procedures and attitudes and will support future actions to standardization and, therefore, harmonization and comparability.

## Abbreviations

ACAAI: American College of Allergy, Asthma and Immunology
AAAAI: American Academy of Allergy Asthma and Immunology
AME: Africa/Middle-East
AP: Asia-Pacific
EAACI: European Academy of Allergy and Clinical Immunology
ENDA: European Network of Drug Allergy
EU: Europe
LA: Latin-America
NA: North America
PT: Skin patch test
WAO: World Allergy Organization
WAO JM: WAO Junior Members
